# Identification of *Cannabis sativa* L. (hemp) Retailers by Means of Multivariate Analysis of Cannabinoids

**DOI:** 10.3390/molecules24193602

**Published:** 2019-10-07

**Authors:** Sara Palmieri, Marcello Mascini, Antonella Ricci, Federico Fanti, Chiara Ottaviani, Claudio Lo Sterzo, Manuel Sergi

**Affiliations:** Faculty of Bioscience and Technology for Food, Agriculture and Environment, University of Teramo, 64100 Teramo, Italy; spalmieri@unite.it (S.P.); aricci@unite.it (A.R.); ffanti@unite.it (F.F.); cottaviani@unite.it (C.O.); closterzo@unite.it (C.L.S.)

**Keywords:** *Cannabis sativa* L., HPLC-MS/MS analysis, cannabinoids, multivariate analysis, partial least squares discriminant analysis (PLS-DA)

## Abstract

In this work, the concentration of nine cannabinoids, six neutral cannabinoids (THC, CBD, CBC, CBG, CBN and CBDV) and three acidic cannabinoids (THCA CBGA and CBDA), was used to identify the Italian retailers of *Cannabis sativa* L. (hemp), reinforcing the idea that the practice of categorizing hemp samples only using THC and CBD is inadequate. A high-performance liquid chromatography/high-resolution mass spectrometry (HPLC-MS/MS) method was developed for screening and simultaneously analyzing the nine cannabinoids in 161 hemp samples sold by four retailers located in different Italian cities. The hemp samples dataset was analyzed by univariate and multivariate analysis with the aim to identify the hemp retailers without any other information on the hemp samples like *Cannabis* strains, seeds, soil and cultivation characteristics, geographical origin, product storage, etc. The univariate analysis highlighted that the hemp samples could not be differentiated by using any of the nine cannabinoids analyzed. To evaluate the real efficiency of the discrimination among the four hemp retailers a partial least squares discriminant analysis (PLS-DA) was applied. The PLS-DA results showed a very good discrimination between the four hemp retailers with an explained variance of 100% and low classification errors in both calibration (5%) and cross validation (6%). A total of 92% of the hemp samples were correctly classified by the cannabinoid variables in both fitting and cross validation. This work contributed to show that an analytical method coupled with multivariate analysis can be used as a powerful tool for forensic purposes.

## 1. Introduction

In the last years, *Cannabis sativa* L. has been one of the most studied plant over the world [1]. *Cannabis sativa* L is a chemically complex plant which contains several classes of natural compounds, e.g., flavonoids, mono- and sesquiterpenes, steroids, nitrogenous compounds and cannabinoids, associated with medicinal properties of the plant [2,3].

The main cannabinoid constituents are Δ^9^-tetrahydrocannabinol (THC), that possess significant psychotropic properties, and other compounds with less or no psychotropic activity including neutral cannabinoids (cannabidiol CBD, cannabigerol CBG, cannabichromene CBC, cannabinol CBN, cannabidivarin CBDV) and acidic cannabinoids (tetrahydrocannabinolic acid THCA, cannabidiolic acid CBDA and cannabigerolic acid CBGA). The species *sativa* is recognized as monotypic classification, that can be divided into different chemotypes based on the specific cannabinoid profile [4,5]. 

The interest in *Cannabis sativa* L. has increased in Italy mainly due to the latest legislation (N. 242 of 2 December 2016); the concentration of THC is used to classify two types of *Cannabis sativa* L. a fiber-type plant with low content of THC (<0.2% w/w) and a prohibited drug-type plant where the content of THC is >0.6% w/w [6]. There are hundreds of hemp strains available in the marketplace based on aroma, plant size, different cultivation, characteristic of the soil and overall yield [4,7,8]. Therefore, there is a request to develop cost effective and easy-to-use quantitative and qualitative methods for the identification and classification of hemp products [6].

The THC and CBD are the reference cannabinoids to extrapolate the phytochemical composition of the hemp, but different works have proved that that strains with similar THC/CBD content have different effects on human physiology [9,10,11].

The present work focused on the chemical content of cannabinoids as a convenient tool to identify the hemp retailers without any other information on the hemp samples like *Cannabis* strains, seeds, soil and cultivation characteristics, geographical origin, product storage, etc. Moreover, the results of this work supported the thesis that the only concentrations of THC and CBD are not enough to discriminate commercial hemp [9]. The analysis of cannabinoids was carried out by means of HPLC-MS/MS, overcoming the phytocannabinoid decomposition due to the heating used to identify and quantify cannabinoids using methods like GC [12,13].

PLS-DA chemometric approach identified correctly the four hemp retailers by using the nine cannabinoids analytical profile of 161 hemp samples, showing that an analytical method coupled with multivariate analysis can lead to a powerful tool for forensic purposes. Chemometric models have already been used to understand phytochemical diversity showing the advantages of multivariate analysis [9,14].

A number of works in the literature reported the determination of THC and CBD concentration in hemp samples [8,15], however to the best of our knowledge, there is a lack of information regarding the evaluation of the comprehensive cannabinoid profile in hemp products with HPLC-MS/MS [7,16,17].

## 2. Results and Discussion

The dataset used for both univariate or multivariate analysis (Table S1, in Supplementary Material) was composed of 161 hemp samples sampled in 2018 from four Italian retailers, and the concentration of the nine cannabinoids, the six neutral cannabinoids (THC, CBD, CBC, CBG, CBN and CBDV) and the three acidic cannabinoids (THCA CBGA and CBDA).

As reported in Table 1, the hemp samples were sold in four Italian cities from three Italian regions. The hemp samples were sold without reporting information like *Cannabis* strains, seeds, soil and cultivation characteristics, geographical origin, product storage, etc.

Data vectors belonging to the same hemp retailer were firstly evaluated by the analysis of variance via the graphical representation of box and whisker plot, and afterwards, by two multivariate techniques, the unsupervised principal component analysis (PCA) and supervised PLS-DA.

**Figure 1:**
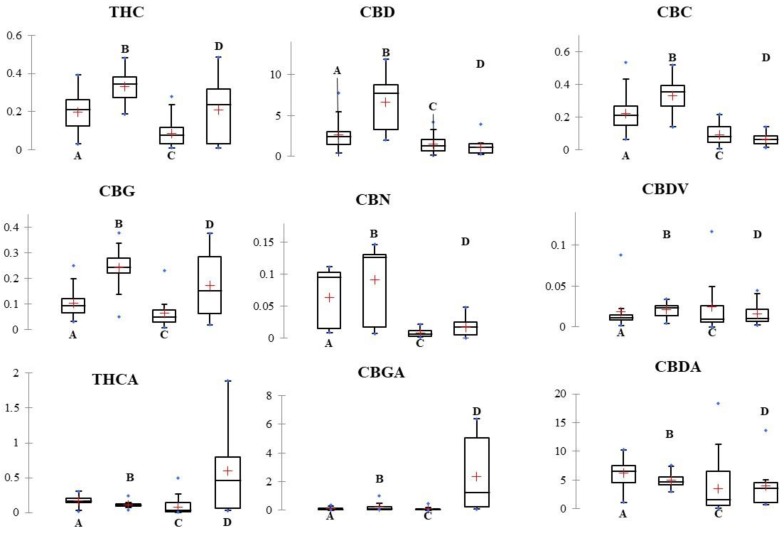
Box and whisker plot of the relative concentrations of the six neutral (THC, CBD, CBC, CBG, CBN and CBDV) and the three acidic cannabinoids (THCA CBGA and CBDA) in the 161 hemp samples. The hemp samples were grouped as sold by the four Italian hemp retailers. Concentration of cannabinoids was reported as % w/w. Y axis title = Concentration (% w/w). X axis Title = Hemp retailers. Median and average were depicted with a flat black line and a red cross, respectively.

As shown in Figure 1, the use of the nine cannabinoids variables led to no statistical differences between the hemp samples grouped as sold by the hemp retailers. Except of the high average concentration of THCA and CBGA found in the retailer D, the cannabinoids analytical profile was in all cases the same with high average concentrations of CBD and CBDA and low average concentrations of the other seven cannabinoids. CBN average concentration was found particularly high in A and B retailers. Interestingly, CBN is an oxidation product of THCA and the high content of this cannabinoid can also be a marker of inflorescence quality. The average concentration of THC was in all cases below of 0.35% w/w.

The results of Tukey HSD multiple comparison test reported in Supplementary Material (Table S2) showed that the acidic cannabinoids had a lower capacity of discrimination than their neutral forms. In fact, the neutral cannabinoids showed a partial discrimination between the four hemp retailers, apart from CBDV.

The univariate data analysis highlighted that the hemp samples sold by the four retailers could not be differentiated only using the analysis of variance because of the overlap in cannabinoids composition of the hemp retailers.

The correlation between the nine cannabinoids analyzed in the hemp samples dataset was analyzed by computing the Pearson coefficients (Table 2). The data showed a partial positive correlation between neutral cannabinoids especially between THC, CBD, and CBC. The only neutral cannabinoid without any correlation was CBDV that belongs to a class of cannabinoids synthesized from a different precursor and structurally different from the classical cannabinoids. No correlations were observed between the six neutral and two acidic cannabinoids THCA and CBGA or within the acidic forms. Only a weak correlation between CBG and CBGA could be remarked as reported also by other work, due to the decarboxylation of CBGA that produces not only CBG but also the other cannabinoids [18]. The acidic cannabinoid CBDA slight correlated with all the six neutral cannabinoids. 

These results agreed with data reported by other works and they can be explained by the biosynthesis of cannabinoids [19,20,21].

This partial correlation between variables provided suitable contributions when treated with multivariate statistical procedures.

All data obtained with HPLC-MS/MS analysis were processed by PCA to find every possible cluster within the hemp samples dataset in an unsupervised way. Before applying the PCA algorithm, data were linearly normalized and auto-scaled (zero mean and unitary variance) in order to remove differences in concentration range. PCA was applied to inspect the multivariate data structure by decomposing the data matrix of the 161 hemp samples (in the matrix rows) and the nine cannabinoids analyzed (the matrix columns).

**Figure 2:**
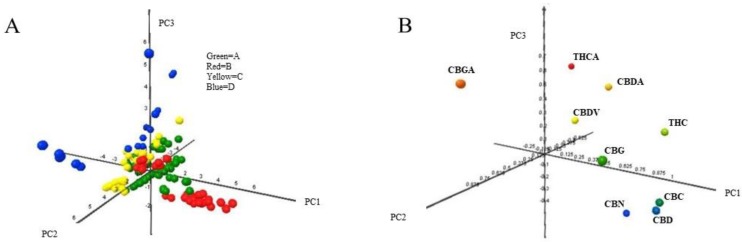
Scores plot (**A**) and loadings plot (**B**) obtained from the PCA on the matrix data of the 161 hemp samples (in the matrix rows) and the nine cannabinoids analyzed (the matrix columns). Plots of the first three components (explained variance: PC1 = 42.7%; PC2 = 15.8%; PC3 = 14.9%; total = 73.4%). Data have been auto-scaled (zero mean and unitary variance) before PCA. The four Hemp retailers are marked with different colors: Green = A; Red = B; Yellow = C; Blue = D.

Figure 2 depicted the scores and loading plots of the first three principal components. The first component represented 42.7% of the variance, the second 15.8% and the third 14.9% accounting 74.4% of the total variance for the first three principal components.

The score points (Figure 2A), representing the new coordinates of the hemp samples, were interpreted assuming that close distance in plot plane is a measure of the similitude between samples. PC 1 separated well both hemp samples sold by retailers B and D but clustered in the cartesian graph origin the hemp samples sold by the retailers A and C. PC 2 did not influence the hemp samples from retailers B and D but it contributed to the dispersion within the hemp samples sold by retailers A and C highlighting a similar variance behavior of those two retailers that started to be separated only along the PC 3 axis that spread both samples sold by retailers B and D.

The loadings (Figure 2B), representing the contribution of each cannabinoid to the principal components, contributed mostly to the hemp samples separation on the PC 1 and PC 3. The PC 1 axis highlighted the differences among neutral and acidic cannabinoids. Neutral cannabinoids contributed significantly to the separation of the hemp samples sold by retailers B. On the other hand, the acid cannabinoid CBGA played an important role in separation of the hemp samples sold by retailer D. CBN, CBD and CBC had very similar pattern recognition performance contributing only in separating hemp samples on PC 1. The hemp samples sold by retailers A and C were influenced by THCA, CBDA and CBDV that contributed to the dispersion on PC 2 and to the separation on PC3.

The unsupervised PCA algorithm could partially discriminate hemp samples sold by the four Italian retailers.

To evaluate the real efficiency of the discrimination among the four retailers a supervised multivariate discriminant analysis was applied. The dataset of 161 hemp samples and nine cannabinoids concentration was used for this approach (Table S1). As in any supervised classification techniques, the classes must be chosen a priori. The choice for the hemp samples in this case was to choose the hemp retailers as classes. With this classification scheme, a PLS-DA model has been built. PLS-DA is an extension of PLS, by projecting intercorrelated X-variables from high dimensional space into low-dimensional space according to a Y-vector that encodes the class membership in a set of categorized variables (1 and 0 values, respectively) [22,23]. A numerical evaluation of the classification properties was obtained by considering the cross validation of the PLS-DA method according to the ‘venetian blinds’ technique.

The model was evaluated using the following parameters: component in model, explained variance in percentage, error rate in calibration and in cross-validation. Moreover, specificity sensitivity and precision of the four classes, corresponding to the four Italian hemp retailers, were computed in fitting and cross-validation.

The statistical summary results of the PLS-DA algorithm were reported in Table 3. The results showed a very good discrimination between the four classes with an explained variance of 100% and low classification errors in both calibration (5%) and cross validation (6%) by using eight model components previously optimized by the algorithm.

Retailers C and D had the highest sensitivity respectively for fitting and cross-validation, contributing to low classification errors. A very good sensitivity and precision in both calibration and cross-validation were found for retailers A, B and D.

Real-predicted samples were reported in Table 4 using confusion matrix format. A total of 92% of the samples have been correctly classified by cannabinoids variables in fitting and cross validation. In fitting, retailers C and D showed 100% and 94% of correspondences between real and predicted samples. In cross-validation, this percentage decreased to 95% for retailer C and increased to 100% for retailer D. The retailers A and B had the highest percentage of misclassified samples, with all misclassified samples assigned to retailer C. Those results underlined a close analytical cannabinoids profile between the retailers A, B and C, also highlighted by the PCA model. The class assigned to each of the 161 hemp samples by the PLS-DA model in fitting and cross validation was reported in Supplementary Material (Table S3).

## 3. Materials and Methods 

### 3.1. Sample Collection

A total of 161 samples of cannabis were purchased from four Italian retailers labeled A, B, C and D. The samples were bought as whole flowers in 5, 10 or 32-g packages and stored at room temperature until use. All samples were in small zip-lock bags, as typically provided by shops.

### 3.2. Solvents and Chemicals

Methanol, acetonitrile and water were HPLC grade and were obtained from VWR (Milan, Italy). Formic acid (98%) LC-MS grade and cannabinoid standards of the six neutral cannabinoids (THC, CBD, CBC, CBG, CBN and CBDV), the three acidic cannabinoids (THCA CBGA and CBDA) and internal standard (IS) tetrahydrocannabinol deuterated (Δ^9^-THC-D_3_) were purchased from Sigma-Aldrich (Steinheim, Germany). All standards were provided as 1.0 mg/mL solutions in methanol. Ethanol absolute anhydrous (>99%) was obtained from CARLO ERBA (Milan, Italy).

### 3.3. Sample Preparation

The extracts were prepared using the procedure of Wang et al. (2018) with slight changes [13]. Briefly, before the extraction, every sample was homogenized. Dried sample was triturated for 10 sec with a chopper (Kenwood Quad Blade CH580 Chopper) for 3 times, then crushed with mortar, and finally sift with a 1 mm sieve. Fine powder of plant material (10 mg) was accurately weighed into a 1.5 mL Eppendorf vial and extracted with 1 mL of ethanol in an ultrasonic water bath for 30 min, followed by centrifugation at room temperature at 10,000 rpm for 15 min. Prior to HPLC analysis, the supernatant was passed through a 0.2 µm PTFE filter, diluted 2000 times and collected in an HPLC vial.

### 3.4. HPLC-MS/MS Analysis

All analyses were performed on a Nexera LC20AD XR system, with Prominence 20AD autosampler. The system was equipped with a vacuum degasser and column oven coupled with a 4500 Qtrap from Sciex (Concord, ON, Canada) equipped with a Turbo V electrospray ionization (ESI) source. For analysis of the nine cannabinoids of this work, we used a Kinetex C18-XB column (100 × 2.1 mm ID) from Phenomenex (Torrance, CA, USA) packed with core-shell particles of 2.6 μm held at a temperature of 35 °C. The mobile phase consisted of water containing 5 mM formic acid (phase A) and acetonitrile with 5 mM formic acid (phase B). Analysis was performed using the following gradient elution at a flow rate of 0.3 mL/min: from 0 to 1.1 min gradient was held at 70% of phase B; from 1.1 to 2 min phase B was increased from 70% to 90%; at 3 min, concentration of phase B was 99% and was held for 2 min. Gradient returned to initial condition in 0.2 min, followed by a 2.8 min equilibration, in a total run time of 8 min. The injection volume was 3 μL and all samples were injected in triplicate. The analytes were detected in positive ionization (PI) with a capillary voltage of 5500 V, using air ion source gas at 60 psi and nitrogen ion source gas at 40 psi at a temperature of 500 °C. Two multi-reaction monitoring (MRM) transitions were chosen for each analyte. All source and instrument parameters for the monitored analytes were tuned by injecting each single standard solution at a concentration of 10 ng/μL at 7 μL/min by a syringe pump. The ion currents were acquired in MRM mode and quantitation was performed by the IS method by means of Multiquant Software from Sciex (Concord, ON, Canada) using THC-D_3_. All samples were analyzed in triplicate. The selected MRM transitions and HPLC–MS/MS parameters are reported in Table S4 in Supplementary Materials.

### 3.5. Analytical Procedure Validation

Limit of quantification (LOQ), limit of detection (LOD), linearity, precision and accuracy were evaluated in the analytical procedure validation. For each analyte a calibration curve was built with 11 points, repeated in triplicate (0.1–250.0 ng mL^−1^) of standard solutions. For each concentration level, injections were performed in triplicate and the average value was used for the external standard calibration curves. Because of the absence of “blank” matrices, the LOD and LOQ were obtained at the signal-to-noise ratios of 3:1 and 10:1, respectively, by analyzing different diluted standard samples. For intraday relative standard deviation (RSD) was considered one-day measures of six sample replicates (intraday precision or repeatability), whereas for interday RSD, samples were analyzed for three consecutive days and twice each day (interday precision or with reproducibility) at three different standard concentrations. The results of the validation experiments are shown in the Tables S5–S7.

### 3.6. Statistical Analysis

Univariate analysis was performed using XLSTAT software (Addinsoft, Long Island City, NY, USA). Experimental results were expressed as means ± standard deviations. Statistical significance was assessed using analysis of variance (ANOVA) with the Tukey HSD (honestly significant difference) multiple comparison analysis. The criterion for statistical significance of differences was *P* < 0.05 for all comparisons.

Multivariate statistical analysis was performed using two different approaches, PCA and PLS-DA by means of MatLab R2011b (Mathworks, Natick, MA, USA) integrated with two toolboxes for MatLab obtained from Milano Chemometrics and QSAR Research Group [24,25]. The data set consisted of 161 × 9, in which rows represented the samples (161 hemp samples), and columns the nine cannabinoids variables. Data have been auto-scaled (zero mean and unitary variance). Data vectors belonging to the same retailer were firstly analyzed by unsupervised PCA. This technique gives the possibility to project data from a higher to a lower dimensional space having a data overview without any preliminary assumptions [26]. Then, the supervised technique PLS-DA was applied to the auto-scaled data matrix of the nine cannabinoids profiles in order to improve the separation between hemp retailers. PLS-DA was used as a supervised deterministic classification technique capable of discriminating the observations on the basis of a class membership categorical matrix [27,28]. PLS-DA was performed on the dataset using also a cross validation of the model by using ‘venetian blinds’ technique with number of cv groups equal to 3. Using confusion matrices, the reliability of the classification models achieved was studied in terms of recognition ability (percentage of the members of the training set correctly classified) and prediction ability (percentage of the members of the test set correctly classified using the rules developed in the training step).

## 4. Conclusions

The analysis of nine cannabinoids by means of HPLC-MS/MS was used to evaluate the feasibility to identify hemp retailers located in Italy without having any other information on the hemp sold like *Cannabis* strain, soil and cultivation characteristics, geographical origin, product storage, etc.

The cannabinoids analytical profile was, in all cases, the same with high concentrations of CBD and CBDA and low concentrations of the other seven cannabinoids. The univariate evaluation of the cannabinoids showed no statistical differences between the hemp samples, demonstrating that analyzing each cannabinoid individually it was not enough to identify the hemp retailers.

On the other hand, the synergic contribution of the nine cannabinoids concentrations could identify the hemp retailers as demonstrated by PLS-DA algorithm. A total of 92% of the hemp samples were correctly classified by the cannabinoid variables in both fitting and cross validation. 

In conclusion, the present study contributes to the characterization of hemp samples sold by retailers without any other information, proving that a simple chemical analysis coupled with a robust chemometric method could be a powerful tool for forensic purposes.

## Figures and Tables

**Table 1:** The summary of the dataset used in this work. The 161 hemp samples were classified as sold by the four Italian retailers. Region and city origin of retailers were also reported.

Retailer	№ of Samples	Region	City	Label
A	63	Lombardy	Milan	A1–63
B	43	Lombardy	Mantova	B1–43
C	38	Lazio	Pomezia	C1–38
D	17	Abruzzo	Tortoreto	D1–17

**Table 2:** Correlation matrix (Pearson coefficients) between the nine cannabinoids variables (THC, CBD, CBC, CBG, CBN, CBDV, THCA CBGA and CBDA). The correlation coefficients were calculated using the relative concentrations of cannabinoids in the 161 hemp samples sold by the four Italian retailers.

Cannabinoids	THC	CBD	CBC	CBG	CBN	CBDV	THCA	CBGA	CBDA
**THC**	**1.00**	0.80	0.81	0.61	0.50	0.19	0.34	−0.16	0.46
**CBD**	0.80	**1.00**	0.91	0.68	0.64	0.20	−0.17	−0.17	0.21
**CBC**	0.81	0.91	**1.00**	0.65	0.60	0.19	−0.19	−0.23	0.35
**CBG**	0.61	0.68	0.65	**1.00**	0.31	0.12	−0.17	0.44	0.19
**CBN**	0.50	0.64	0.60	0.31	**1.00**	−0.03	−0.03	−0.25	0.11
**CBDV**	0.19	0.20	0.19	0.12	−0.03	**1.00**	−0.01	−0.04	0.19
**THCA**	0.34	−0.17	−0.19	−0.17	−0.03	−0.01	**1.00**	0.01	0.18
**CBGA**	−0.16	−0.17	−0.23	0.44	−0.25	−0.04	0.01	**1.00**	−0.04
**CBDA**	0.46	0.21	0.35	0.19	0.11	0.19	0.18	−0.04	**1.00**

**Table 3:** PLS-DA classification results in fitting and cross-validation. Cross-validation ‘venetian blinds’ technique was used with the number of cv groups equal to 3. The optimal components for the model was previously calculated using the MatLab toolbox from Milano Chemometrics and Quantitative Structure Activity Relationship (QSAR) Research Group.

**PLS-DA results**			
**samples**	161		
**variables**	9		
**classes**	4		
**component in model**	8		
**explained variance (%)**	100%		
**error rate CL ^1^**	0.05		
**error rate CV ^2^**	0.06		
**Retailer**	**Specificity**	**Sensitivity**	**Precision**
	**Fitting**		
**A**	1.00	0.86	1.00
**B**	1.00	0.88	1.00
**C**	0.88	1.00	0.72
**D**	1.00	0.94	1.00
	**Cross-Validation**		
**A**	1.00	0.86	1.00
**B**	0.98	0.88	0.95
**C**	0.89	0.95	0.72
**D**	1.00	1.00	1.00

^1^ CL= calibration; ^2^ CV= cross-validation.

**Table 4:** Confusion matrix of the PLS-DA classification model (fitting and validation results are both reported). Cross validation ‘venetian blinds’ technique was used with the number of cv groups equal to 3. True classes are read along the columns and estimated classes along the rows. The total accuracy was also reported.

**Fitting**					
**real/predicted**	**A**	**B**	**C**	**D**	
**A**	54	0	9	0	86%
**B**	0	38	5	0	88%
**C**	0	0	38	0	100%
**D**	0	0	1	16	94%
				**Total**	**92%**
**Cross-Validation**					
**real/predicted**	**A**	**B**	**C**	**D**	
**A**	54	0	9	0	86%
**B**	0	38	5	0	88%
**C**	0	2	36	0	95%
**D**	0	0	0	17	100%
				**Total**	**92%**

## Supplementary Materials

The following are available online, Table S1: Relative concentrations of the nine cannabinoids in the 161 hemp samples grouped as sold by the four Italian hemp retailers. Region and city origin of the retailers were also reported. In bold the descriptive statistics of each retailer group. CV = coefficient of variation; SD = standard deviation. Concentration of cannabinoids was reported as % w/w; Table S2: Analysis of variance (ANOVA) using the Tukey HSD (honestly significant difference) multiple comparison test. The criterion for statistical significance of differences was *P* < 0.05 for all comparisons; Table S3: Comparison between retailer assigned classes (A) and classes calculated by the PLS-DA algorithm using fitting (F) and cross validation (V). The PLS-DA model calculated the classes using the relative concentrations of the nine cannabinoids in the 161 hemp samples and, as classes, the four hemp retailers; Table S4: MS/MS parameters. Multi-reaction monitoring (MRM) transitions: Q1 MASS = precursor ion mass (amu); Q3 MASS = product ion mass (amu). TIME = dwell time. DP (V) = de-clustering potential (amu). EP (V) = entrance potential. CE (V) = collision energy. CXP (V) = cell exit potential; Table S5: Limit of quantification (LOQ), limit of detection (LOD), calibration curve equation and correlation coefficient obtained in analytical procedure validation; Table S6: The accuracy results, reported in percentage (%), obtained in analytical procedure validation; Table S7: The intraday interday precision results, reported in percentage (%), obtained in analytical procedure validation.
