# Supplementary material for: Identification of Cannabis sativa L. (hemp) Retailers by Means of Multivariate Analysis of Cannabinoids
Source: Molecules. 2019 Oct 7;24(19):3602. doi: 10.3390/molecules24193602 (PMC6804059; doi:10.3390/molecules24193602)
Supplement: Supplementary file 1 [file molecules-24-03602-s001.zip › molecules-596134-SI.pdf]

Table S1. Relative concentrations of the nine cannabinoids in the 161 hemp samples grouped as sold by the four Italian hemp retailers. Region and city origin of the retailers were also reported. In bold the descriptive statistics of each retailer group. CV = coefficient of variation; SD = standard deviation. Concentration of cannabinoids was reported as % w/w.

Retailer	Region	City	Label	THC	CBD	CBC	CBG	CBN	CBDV	THCA	CBGA	CBDA
A	Lombardy	Mantova	A 1	0.09	0.98	0.08	0.04	0.01	0.00	0.16	0.11	5.93
A	Lombardy	Mantova	A 2	0.09	0.95	0.06	0.04	0.01	0.01	0.16	0.10	6.00
A	Lombardy	Mantova	A 3	0.13	1.19	0.10	0.05	0.01	0.01	0.22	0.15	7.48
A	Lombardy	Mantova	A 4	0.13	1.19	0.09	0.05	0.01	0.00	0.21	0.14	7.33
A	Lombardy	Mantova	A 5	0.20	2.36	0.20	0.10	0.01	0.00	0.14	0.16	6.73
A	Lombardy	Mantova	A 6	0.20	2.63	0.21	0.10	0.01	0.01	0.14	0.18	7.47
A	Lombardy	Mantova	A 7	0.15	2.42	0.19	0.07	0.01	0.00	0.10	0.08	5.69
A	Lombardy	Mantova	A 8	0.16	2.37	0.20	0.07	0.01	0.00	0.10	0.08	5.67
A	Lombardy	Mantova	A 9	0.20	2.23	0.15	0.04	0.01	0.01	0.15	0.12	6.15
A	Lombardy	Mantova	A 10	0.22	2.41	0.23	0.05	0.02	0.01	0.16	0.13	6.80
A	Lombardy	Mantova	A 11	0.13	1.28	0.12	0.03	0.01	0.00	0.13	0.10	5.19
A	Lombardy	Mantova	A 12	0.14	1.56	0.13	0.04	0.02	0.00	0.15	0.12	6.15
A	Lombardy	Mantova	A 13	0.35	7.73	0.54	0.25	0.02	0.02	0.02	0.15	5.53
A	Lombardy	Mantova	A 14	0.26	5.65	0.38	0.18	0.02	0.02	0.02	0.11	4.09
A	Lombardy	Mantova	A 15	0.27	5.90	0.41	0.18	0.02	0.01	0.02	0.11	4.04
A	Lombardy	Mantova	A 16	0.27	5.68	0.38	0.18	0.02	0.02	0.01	0.10	3.83
A	Lombardy	Mantova	A 17	0.32	5.48	0.43	0.20	0.02	0.02	0.05	0.21	6.53
A	Lombardy	Mantova	A 18	0.30	5.14	0.39	0.19	0.02	0.01	0.06	0.19	6.35
A	Lombardy	Mantova	A 19	0.39	6.49	0.49	0.24	0.02	0.02	0.04	0.18	6.47
A	Lombardy	Mantova	A 20	0.37	6.75	0.48	0.25	0.02	0.02	0.04	0.21	6.58
A	Lombardy	Mantova	A 21	0.13	1.62	0.16	0.07	0.10	0.01	0.19	0.02	4.69
A	Lombardy	Mantova	A 22	0.16	2.02	0.18	0.08	0.10	0.01	0.22	0.02	5.61
A	Lombardy	Mantova	A 23	0.29	3.10	0.28	0.11	0.11	0.01	0.26	0.05	9.52
A	Lombardy	Mantova	A 24	0.07	0.94	0.12	0.06	0.10	0.01	0.14	0.01	2.61
A	Lombardy	Mantova	A 25	0.10	1.23	0.14	0.07	0.10	0.01	0.17	0.02	3.51
A	Lombardy	Mantova	A 26	0.03	0.44	0.09	0.05	0.09	0.01	0.12	0.00	1.17
A	Lombardy	Mantova	A 27	0.28	3.19	0.28	0.11	0.11	0.01	0.30	0.03	9.44
A	Lombardy	Mantova	A 28	0.07	0.88	0.12	0.06	0.10	0.01	0.14	0.01	2.42
A	Lombardy	Mantova	A 29	0.03	0.43	0.09	0.05	0.10	0.01	0.12	0.01	1.09
A	Lombardy	Mantova	A 30	0.18	2.25	0.20	0.09	0.10	0.01	0.20	0.03	6.85
A	Lombardy	Mantova	A 31	0.11	1.40	0.16	0.07	0.10	0.01	0.18	0.02	4.09
A	Lombardy	Mantova	A 32	0.09	1.07	0.13	0.06	0.10	0.01	0.15	0.02	3.16
A	Lombardy	Mantova	A 33	0.04	0.43	0.10	0.05	0.09	0.01	0.12	0.01	1.19
A	Lombardy	Mantova	A 34	0.18	2.19	0.21	0.09	0.10	0.01	0.23	0.04	6.46
A	Lombardy	Mantova	A 35	0.09	1.15	0.14	0.06	0.10	0.01	0.16	0.02	3.38
A	Lombardy	Mantova	A 36	0.18	2.16	0.21	0.09	0.10	0.01	0.24	0.04	6.78
A	Lombardy	Mantova	A 37	0.11	1.36	0.15	0.07	0.10	0.01	0.14	0.03	3.89
A	Lombardy	Mantova	A 38	0.12	1.49	0.16	0.07	0.10	0.01	0.20	0.03	4.41
A	Lombardy	Mantova	A 39	0.31	3.74	0.30	0.12	0.11	0.01	0.28	0.05	10.27

A	Lombardy	Mantova	A 40	0.30	3.31	0.28	0.12	0.11	0.01	0.26	0.07	9.09
A	Lombardy	Mantova	A 41	0.24	3.00	0.25	0.11	0.11	0.01	0.22	0.05	8.19
A	Lombardy	Mantova	A 42	0.22	2.39	0.23	0.09	0.10	0.01	0.16	0.04	6.50
A	Lombardy	Mantova	A 43	0.08	0.99	0.13	0.06	0.10	0.01	0.14	0.02	2.54
A	Lombardy	Mantova	A 44	0.14	1.57	0.17	0.07	0.10	0.01	0.17	0.02	4.21
A	Lombardy	Mantova	A 45	0.21	2.63	0.23	0.09	0.10	0.01	0.26	0.05	7.47
A	Lombardy	Mantova	A 46	0.29	3.30	0.27	0.10	0.11	0.01	0.30	0.05	9.74
A	Lombardy	Mantova	A 47	0.22	2.32	0.22	0.09	0.11	0.01	0.22	0.04	7.28
A	Lombardy	Mantova	A 48	0.24	2.83	0.24	0.10	0.11	0.01	0.27	0.04	8.76
A	Lombardy	Mantova	A 49	0.16	1.73	0.17	0.08	0.10	0.01	0.16	0.02	5.44
A	Lombardy	Mantova	A 50	0.21	2.41	0.21	0.09	0.10	0.01	0.23	0.04	7.47
A	Lombardy	Mantova	A 51	0.23	2.91	0.24	0.10	0.11	0.01	0.18	0.03	7.67
A	Lombardy	Mantova	A 52	0.20	2.52	0.21	0.09	0.10	0.01	0.16	0.04	6.68
A	Lombardy	Mantova	A 53	0.28	3.26	0.26	0.11	0.10	0.01	0.21	0.04	7.97
A	Lombardy	Mantova	A 54	0.22	2.67	0.22	0.09	0.10	0.01	0.18	0.04	6.66
A	Lombardy	Mantova	A 55	0.22	2.72	0.24	0.09	0.10	0.01	0.20	0.03	7.37
A	Lombardy	Mantova	A 56	0.25	2.98	0.24	0.14	0.02	0.07	0.21	0.35	8.71
A	Lombardy	Mantova	A 57	0.24	2.90	0.25	0.14	0.02	0.07	0.21	0.36	9.00
A	Lombardy	Mantova	A 58	0.21	2.66	0.23	0.15	0.02	0.06	0.20	0.37	7.80
A	Lombardy	Mantova	A 59	0.22	2.46	0.20	0.14	0.01	0.06	0.20	0.34	7.61
A	Lombardy	Mantova	A 60	0.27	3.04	0.27	0.17	0.01	0.07	0.14	0.36	7.12
A	Lombardy	Mantova	A 61	0.27	3.51	0.29	0.18	0.01	0.09	0.17	0.36	7.87
A	Lombardy	Mantova	A 62	0.24	2.88	0.25	0.12	0.01	0.07	0.16	0.28	7.18
A	Lombardy	Mantova	A 63	0.34	3.60	0.35	0.17	0.01	0.09	0.19	0.36	8.76
			**Samples**	63	63	63	63	63	63	63	63	63
			**Max Value**	0.39	7.73	0.54	0.25	0.11	0.09	0.30	0.37	10.27
			**Min Value**	0.03	0.43	0.06	0.03	0.01	0.00	0.01	0.00	1.09
			**Average**	0.20	2.64	0.22	0.10	0.06	0.02	0.17	0.10	6.15
			**Mode**	0.13	2.63	#N/A	#N/A	#N/A	0.01	#N/A	#N/A	#N/A
			**Median**	0.21	2.41	0.21	0.09	0.10	0.01	0.16	0.05	6.53
			**SD**	0.09	1.60	0.10	0.05	0.04	0.02	0.07	0.11	2.18
			**CV**	0.44	0.61	0.46	0.52	0.70	1.16	0.42	1.04	0.35

Retailer	Region	City	Label	THC	CBD	CBC	CBG	CBN	CBDV	THCA	CBGA	CBDA
B	Lombardy	Milano	B 1	0.20	2.11	0.14	0.05	0.01	0.00	0.24	0.16	7.48
B	Lombardy	Milano	B 2	0.19	2.08	0.15	0.05	0.01	0.00	0.22	0.16	7.38
B	Lombardy	Milano	B 3	0.23	2.28	0.21	0.31	0.02	0.01	0.19	0.99	5.80
B	Lombardy	Milano	B 4	0.21	2.18	0.20	0.27	0.01	0.01	0.18	0.97	5.51
B	Lombardy	Milano	B 5	0.23	2.46	0.21	0.28	0.02	0.01	0.10	0.79	4.65
B	Lombardy	Milano	B 6	0.31	3.18	0.29	0.37	0.02	0.02	0.12	1.02	6.34
B	Lombardy	Milano	B 7	0.38	3.67	0.27	0.29	0.02	0.01	0.14	0.53	7.54
B	Lombardy	Milano	B 8	0.38	3.68	0.27	0.28	0.02	0.01	0.14	0.50	7.56
B	Lombardy	Milano	B 9	0.34	3.29	0.28	0.29	0.02	0.01	0.12	0.61	6.25
B	Lombardy	Milano	B 10	0.30	3.22	0.26	0.30	0.01	0.01	0.12	0.60	6.09
B	Lombardy	Milano	B 11	0.20	2.60	0.16	0.14	0.01	0.02	0.11	0.25	5.22

B	Lombardy	Milano	B 12	0.22	2.54	0.18	0.12	0.01	0.03	0.11	0.22	5.25
B	Lombardy	Milano	B 13	0.23	2.23	0.18	0.17	0.01	0.01	0.14	0.48	5.45
B	Lombardy	Milano	B 14	0.19	1.99	0.17	0.15	0.01	0.01	0.13	0.45	4.85
B	Lombardy	Milano	B 15	0.31	7.16	0.35	0.24	0.13	0.02	0.13	0.07	3.77
B	Lombardy	Milano	B 16	0.25	5.87	0.30	0.20	0.12	0.02	0.10	0.06	3.23
B	Lombardy	Milano	B 17	0.41	9.38	0.42	0.30	0.14	0.03	0.10	0.09	4.81
B	Lombardy	Milano	B 18	0.31	7.97	0.37	0.26	0.13	0.02	0.10	0.08	4.17
B	Lombardy	Milano	B 19	0.48	11.26	0.52	0.38	0.14	0.03	0.10	0.11	5.85
B	Lombardy	Milano	B 20	0.43	10.29	0.48	0.34	0.15	0.03	0.10	0.11	5.53
B	Lombardy	Milano	B 21	0.42	8.27	0.38	0.26	0.13	0.02	0.10	0.09	5.34
B	Lombardy	Milano	B 22	0.34	6.84	0.35	0.23	0.12	0.02	0.11	0.08	4.33
B	Lombardy	Milano	B 23	0.36	7.51	0.36	0.24	0.12	0.02	0.11	0.08	4.56
B	Lombardy	Milano	B 24	0.40	8.29	0.42	0.26	0.13	0.03	0.13	0.09	5.30
B	Lombardy	Milano	B 25	0.24	5.15	0.27	0.18	0.11	0.02	0.09	0.06	2.96
B	Lombardy	Milano	B 26	0.40	8.11	0.38	0.26	0.13	0.03	0.12	0.09	4.74
B	Lombardy	Milano	B 27	0.35	8.01	0.34	0.20	0.13	0.02	0.11	0.05	4.15
B	Lombardy	Milano	B 28	0.38	8.64	0.39	0.21	0.13	0.03	0.08	0.06	4.34
B	Lombardy	Milano	B 29	0.33	8.14	0.38	0.21	0.13	0.03	0.11	0.05	4.35
B	Lombardy	Milano	B 30	0.38	9.42	0.41	0.24	0.14	0.02	0.09	0.07	4.73
B	Lombardy	Milano	B 31	0.38	9.32	0.43	0.23	0.13	0.02	0.09	0.06	4.34
B	Lombardy	Milano	B 32	0.40	9.17	0.42	0.24	0.14	0.03	0.08	0.06	4.54
B	Lombardy	Milano	B 33	0.39	10.36	0.47	0.27	0.13	0.03	0.07	0.08	4.52
B	Lombardy	Milano	B 34	0.38	8.91	0.41	0.25	0.13	0.03	0.10	0.06	3.98
B	Lombardy	Milano	B 35	0.33	8.50	0.40	0.25	0.13	0.03	0.09	0.07	3.86
B	Lombardy	Milano	B 36	0.43	10.48	0.36	0.29	0.14	0.03	0.10	0.07	4.63
B	Lombardy	Milano	B 37	0.48	11.84	0.48	0.32	0.14	0.03	0.03	0.09	5.11
B	Lombardy	Milano	B 38	0.34	7.20	0.36	0.23	0.12	0.02	0.11	0.07	3.79
B	Lombardy	Milano	B 39	0.35	8.18	0.38	0.24	0.12	0.03	0.11	0.06	3.93
B	Lombardy	Milano	B 40	0.35	7.67	0.29	0.23	0.13	0.02	0.10	0.07	3.80
B	Lombardy	Milano	B 41	0.35	7.37	0.37	0.23	0.12	0.02	0.10	0.06	3.90
B	Lombardy	Milano	B 42	0.35	7.70	0.34	0.23	0.13	0.02	0.10	0.06	3.85
B	Lombardy	Milano	B 43	0.38	9.01	0.39	0.27	0.13	0.03	0.05	0.07	4.42
			**Samples**	43	43	43	43	43	43	43	43	43
			**Max Value**	0.48	11.84	0.52	0.38	0.15	0.03	0.24	1.02	7.56
			**Min Value**	0.19	1.99	0.14	0.05	0.01	0.00	0.03	0.05	2.96
			**Average**	0.33	6.59	0.33	0.24	0.09	0.02	0.11	0.23	4.93
			**Mode**	0.23	#N/A	#N/A	#N/A	#N/A	#N/A	0.10	#N/A	#N/A
			**Median**	0.35	7.67	0.36	0.24	0.13	0.02	0.11	0.08	4.65
			**SD**	0.08	3.03	0.10	0.07	0.06	0.01	0.04	0.28	1.13
			**CV**	0.24	0.46	0.30	0.28	0.61	0.40	0.34	1.23	0.23

Retailer	Region	City	Label	THC	CBD	CBC	CBG	CBN	CBDV	THCA	CBGA	CBDA
C	Lazio	Pomezia	C 1	0.28	4.20	0.22	0.23	0.02	0.01	0.26	0.46	18.28
C	Lazio	Pomezia	C 2	0.24	3.12	0.17	0.20	0.02	0.01	0.20	0.42	11.19
C	Lazio	Pomezia	C 3	0.23	3.25	0.16	0.19	0.02	0.00	0.15	0.37	8.80

C	Lazio	Pomezia	C 4	0.20	2.94	0.16	0.18	0.02	0.01	0.17	0.37	7.74
C	Lazio	Pomezia	C 5	0.13	2.29	0.15	0.04	0.01	0.05	0.15	0.10	7.88
C	Lazio	Pomezia	C 6	0.14	2.10	0.14	0.04	0.01	0.05	0.14	0.10	7.41
C	Lazio	Pomezia	C 7	0.17	3.20	0.20	0.07	0.01	0.07	0.21	0.21	10.96
C	Lazio	Pomezia	C 8	0.17	3.07	0.20	0.07	0.01	0.07	0.17	0.22	9.62
C	Lazio	Pomezia	C 9	0.04	0.85	0.06	0.04	0.00	0.01	0.03	0.08	1.55
C	Lazio	Pomezia	C 10	0.05	0.90	0.07	0.04	0.01	0.01	0.03	0.08	1.76
C	Lazio	Pomezia	C 11	0.03	0.60	0.04	0.03	0.00	0.01	0.01	0.04	0.83
C	Lazio	Pomezia	C 12	0.02	0.52	0.04	0.03	0.00	0.01	0.01	0.05	0.73
C	Lazio	Pomezia	C 13	0.05	1.32	0.10	0.03	0.01	0.01	0.03	0.05	2.05
C	Lazio	Pomezia	C 14	0.05	1.26	0.10	0.03	0.01	0.01	0.02	0.04	1.85
C	Lazio	Pomezia	C 15	0.05	0.93	0.05	0.04	0.01	0.09	0.05	0.09	2.30
C	Lazio	Pomezia	C 16	0.07	1.16	0.06	0.05	0.00	0.12	0.05	0.10	2.65
C	Lazio	Pomezia	C 17	0.10	2.14	0.11	0.10	0.01	0.11	0.04	0.10	2.54
C	Lazio	Pomezia	C 18	0.08	1.64	0.08	0.08	0.01	0.09	0.03	0.10	1.98
C	Lazio	Pomezia	C 19	0.12	1.34	0.09	0.08	0.02	0.02	0.05	0.07	1.11
C	Lazio	Pomezia	C 20	0.12	1.63	0.08	0.08	0.02	0.03	0.06	0.07	1.12
C	Lazio	Pomezia	C 21	0.09	1.12	0.06	0.06	0.02	0.02	0.05	0.06	1.00
C	Lazio	Pomezia	C 22	0.10	1.28	0.07	0.07	0.02	0.03	0.04	0.06	0.97
C	Lazio	Pomezia	C 23	0.05	0.93	0.08	0.02	0.00	0.01	0.02	0.03	1.55
C	Lazio	Pomezia	C 24	0.10	0.95	0.06	0.09	0.00	0.00	0.49	0.29	3.78
C	Lazio	Pomezia	C 25	0.09	1.96	0.14	0.07	0.00	0.01	0.01	0.02	0.91
C	Lazio	Pomezia	C 26	0.08	1.94	0.15	0.07	0.01	0.01	0.01	0.02	0.98
C	Lazio	Pomezia	C 27	0.08	2.51	0.17	0.09	0.00	0.01	0.00	0.02	0.58
C	Lazio	Pomezia	C 28	0.06	1.36	0.09	0.06	0.00	0.01	0.01	0.02	0.41
C	Lazio	Pomezia	C 29	0.01	0.24	0.02	0.01	0.00	0.00	0.01	0.01	0.23
C	Lazio	Pomezia	C 30	0.01	0.15	0.01	0.01	0.00	0.00	0.01	0.01	0.13
C	Lazio	Pomezia	C 31	0.02	0.28	0.02	0.01	0.00	0.00	0.01	0.00	0.12
C	Lazio	Pomezia	C 32	0.02	0.29	0.01	0.01	0.00	0.00	0.01	0.00	0.13
C	Lazio	Pomezia	C 33	0.02	0.47	0.03	0.03	0.00	0.01	0.01	0.02	0.43
C	Lazio	Pomezia	C 34	0.02	0.50	0.03	0.02	0.01	0.01	0.01	0.02	0.40
C	Lazio	Pomezia	C 35	0.02	0.48	0.02	0.02	0.00	0.01	0.01	0.02	0.40
C	Lazio	Pomezia	C 36	0.02	0.38	0.03	0.02	0.00	0.01	0.01	0.01	0.32
C	Lazio	Pomezia	C 37	0.10	1.22	0.09	0.05	0.01	0.01	0.16	0.10	8.15
C	Lazio	Pomezia	C 38	0.12	1.30	0.10	0.06	0.01	0.01	0.19	0.12	9.36
			**Samples**	38	38	38	38	38	38	38	38	38
			**Max Value**	0.28	4.20	0.22	0.23	0.02	0.12	0.49	0.46	18.28
			**Min Value**	0.01	0.15	0.01	0.01	0.00	0.00	0.00	0.00	0.12
			**Average**	0.09	1.47	0.09	0.06	0.01	0.02	0.08	0.10	3.48
			**Mode**	#N/A	#N/A	#N/A	#N/A	#N/A	0.00	0.01	#N/A	#N/A
			**Median**	0.08	1.27	0.08	0.05	0.01	0.01	0.03	0.07	1.55
			**SD**	0.07	1.01	0.06	0.05	0.01	0.03	0.10	0.12	4.29
			**CV**	0.78	0.69	0.64	0.85	0.72	1.33	1.32	1.16	1.23

Retailer	Region	City	Label	THC	CBD	CBC	CBG	CBN	CBDV	THCA	CBGA	CBDA
D	Abruzzo	Tortoreto	D 1	0.24	3.43	0.14	0.20	0.03	0.04	0.36	2.14	12.69
D	Abruzzo	Tortoreto	D 2	0.37	3.93	0.14	0.35	0.05	0.04	0.59	5.83	13.59
D	Abruzzo	Tortoreto	D 3	0.17	1.36	0.08	0.15	0.01	0.02	0.32	1.24	4.53
D	Abruzzo	Tortoreto	D 4	0.03	0.36	0.03	0.31	0.01	0.01	0.07	3.30	0.76
D	Abruzzo	Tortoreto	D 5	0.03	0.45	0.02	0.38	0.00	0.01	0.06	3.60	0.90
D	Abruzzo	Tortoreto	D 6	0.46	1.25	0.07	0.07	0.03	0.03	1.85	0.32	4.26
D	Abruzzo	Tortoreto	D 7	0.49	1.11	0.08	0.06	0.02	0.03	1.89	0.35	4.14
D	Abruzzo	Tortoreto	D 8	0.42	0.56	0.04	0.02	0.04	0.00	1.32	0.09	2.91
D	Abruzzo	Tortoreto	D 9	0.32	0.46	0.04	0.02	0.03	0.00	1.03	0.08	2.34
D	Abruzzo	Tortoreto	D 10	0.25	1.60	0.10	0.04	0.02	0.01	0.58	0.13	3.57
D	Abruzzo	Tortoreto	D 11	0.19	1.67	0.09	0.07	0.01	0.02	0.46	0.36	5.03
D	Abruzzo	Tortoreto	D 12	0.30	1.58	0.09	0.07	0.02	0.01	0.79	0.24	4.98
D	Abruzzo	Tortoreto	D 13	0.24	1.24	0.09	0.05	0.02	0.01	0.74	0.18	3.75
D	Abruzzo	Tortoreto	D 14	0.02	0.36	0.04	0.31	0.00	0.01	0.04	6.39	0.93
D	Abruzzo	Tortoreto	D 15	0.01	0.29	0.04	0.25	0.00	0.01	0.03	5.05	0.71
D	Abruzzo	Tortoreto	D 16	0.02	0.37	0.05	0.29	0.00	0.01	0.04	5.23	1.05
D	Abruzzo	Tortoreto	D 17	0.01	0.39	0.05	0.28	0.00	0.01	0.03	5.04	1.00
			**Samples**	17	17	17	17	17	17	17	17	17
			**Max Value**	0.49	3.93	0.14	0.38	0.05	0.04	1.89	6.39	13.59
			**Min Value**	0.01	0.29	0.02	0.02	0.00	0.00	0.03	0.08	0.71
			**Average**	0.21	1.20	0.07	0.17	0.02	0.02	0.60	2.33	3.95
			**Mode**	#N/A	#N/A	#N/A	0.31	0.00	#N/A	#N/A	#N/A	#N/A
			**Median**	0.24	1.11	0.07	0.15	0.02	0.01	0.46	1.24	3.57
			**SD**	0.17	1.06	0.04	0.13	0.01	0.01	0.61	2.39	3.81
			**CV**	0.80	0.89	0.54	0.76	0.86	0.83	1.02	1.03	0.96

Table S2. Analysis of variance (ANOVA) using the Tukey HSD (honestly significant difference) multiple comparison test. The criterion for statistical significance of differences was $P < 0.05$ for all comparisons.

Contrast	Difference	Standardized difference	Pr > Diff	Significant
		**THC**		
C vs B	-0.244	-11.816	**< 0.0001**	Yes
C vs D	-0.122	-4.510	**< 0.0001**	Yes
C vs A	-0.110	-5.750	**< 0.0001**	Yes
A vs B	-0.134	-7.328	**< 0.0001**	Yes
A vs D	-0.012	-0.493	0.961	No
D vs B	-0.122	-4.589	**< 0.0001**	Yes
		**CBD**		
D vs B	-5.393	-9.638	**< 0.0001**	Yes
D vs A	-1.435	-2.688	**0.039**	Yes
D vs C	-0.267	-0.469	0.966	No
C vs B	-5.126	-11.788	**< 0.0001**	Yes
C vs A	-1.168	-2.911	**0.021**	Yes
A vs B	-3.958	-10.245	**< 0.0001**	Yes
		**CBC**		
D vs B	-0.261	-10.303	**< 0.0001**	Yes
D vs A	-0.156	-6.438	**< 0.0001**	Yes
D vs C	-0.022	-0.868	0.821	No
C vs B	-0.239	-12.120	**< 0.0001**	Yes
C vs A	-0.133	-7.334	**< 0.0001**	Yes
A vs B	-0.106	-6.027	**< 0.0001**	Yes
		**CBG**		
C vs B	-0.178	-11.595	**< 0.0001**	Yes
C vs D	-0.108	-5.375	**< 0.0001**	Yes
C vs A	-0.040	-2.788	**0.030**	Yes
A vs B	-0.139	-10.156	**< 0.0001**	Yes
A vs D	-0.069	-3.644	**0.002**	Yes
D vs B	-0.070	-3.536	**0.003**	Yes
		**CBN**		
C vs B	-0.084	-9.311	**< 0.0001**	Yes
C vs A	-0.055	-6.621	**< 0.0001**	Yes
C vs D	-0.009	-0.739	0.881	No
D vs B	-0.075	-6.483	**< 0.0001**	Yes
D vs A	-0.046	-4.187	**0.000**	Yes
A vs B	-0.029	-3.604	**0.002**	Yes
		**CBDV**		
D vs C	-0.009	-1.416	0.492	No
D vs B	-0.005	-0.883	0.814	No
D vs A	-0.003	-0.507	0.957	No
A vs C	-0.006	-1.336	0.541	No

A vs B	-0.002	-0.578	0.938	No
B vs C	-0.003	-0.719	0.889	No
		**THCA**		
C vs D	-0.523	-8.661	< **0.0001**	Yes
C vs A	-0.089	-2.092	0.160	No
C vs B	-0.036	-0.779	0.864	No
B vs D	-0.487	-8.215	< **0.0001**	Yes
B vs A	-0.053	-1.296	0.567	No
A vs D	-0.434	-7.674	< **0.0001**	Yes
		**CBGA**		
A vs D	-2.223	-10.402	< **0.0001**	Yes
A vs B	-0.126	-0.811	0.849	No
A vs C	-0.001	-0.004	1.000	No
C vs D	-2.222	-9.740	< **0.0001**	Yes
C vs B	-0.125	-0.717	0.890	No
B vs D	-2.097	-9.363	< **0.0001**	Yes
		**CBDA**		
C vs A	-2.675	-4.591	< **0.0001**	Yes
C vs B	-1.456	-2.305	0.101	No
C vs D	-0.471	-0.569	0.941	No
D vs A	-2.204	-2.843	**0.026**	Yes
D vs B	-0.985	-1.212	0.620	No
B vs A	-1.219	-2.173	0.135	No

Table S3. Comparison between retailer assigned classes (A) and classes calculated by the PLS-DA algorithm using fitting (F) and cross validation (V). The PLS-DA model calculated the classes using the relative concentrations of the nine cannabinoids in the 161 hemp samples and, as classes, the four hemp retailers.

**A**				**B**				**C**				**D**			
**Label**	**A**	**F**	**V**	**Label**	**A**	**F**	**V**	**Label**	**A**	**F**	**V**	**Label**	**A**	**F**	**V**
A 1	1	3	3	B 1	**2**	3	3	C 1	3	3	2	D 1	4	4	4
A 2	1	3	3	B 2	**2**	3	3	C 2	3	3	3	D 2	4	4	4
A 3	1	3	3	B 3	**2**	2	2	C 3	3	3	3	D 3	4	4	4
A 4	1	3	3	B 4	**2**	2	2	C 4	3	3	2	D 4	4	4	4
A 5	1	1	1	B 5	**2**	2	2	C 5	3	3	3	D 5	4	4	4
A 6	1	1	1	B 6	**2**	2	2	C 6	3	3	3	D 6	4	4	4
A 7	1	3	3	B 7	**2**	2	2	C 7	3	3	3	D 7	4	4	4
A 8	1	1	1	B 8	**2**	2	2	C 8	3	3	3	D 8	4	4	4
A 9	1	3	3	B 9	**2**	2	2	C 9	3	3	3	D 9	4	4	4
A 10	1	1	1	B 10	**2**	2	2	C 10	3	3	3	D 10	4	4	4
A 11	1	3	3	B 11	**2**	3	3	C 11	3	3	3	D 11	4	3	4
A 12	1	3	3	B 12	**2**	3	3	C 12	3	3	3	D 12	4	4	4
A 13	1	1	1	B 13	**2**	2	2	C 13	3	3	3	D 13	4	4	4
A 14	1	1	1	B 14	**2**	3	3	C 14	3	3	3	D 14	4	4	4
A 15	1	1	1	B 15	**2**	2	2	C 15	3	3	3	D 15	4	4	4
A 16	1	1	1	B 16	**2**	2	2	C 16	3	3	3	D 16	4	4	4
A 17	1	1	1	B 17	**2**	2	2	C 17	3	3	3	D 17	4	4	4
A 18	1	1	1	B 18	**2**	2	2	C 18	3	3	3				
A 19	1	1	1	B 19	**2**	2	2	C 19	3	3	3				
A 20	1	1	1	B 20	**2**	2	2	C 20	3	3	3				
A 21	1	1	1	B 21	**2**	2	2	C 21	3	3	3				
A 22	1	1	1	B 22	**2**	2	2	C 22	3	3	3				
A 23	1	1	1	B 23	**2**	2	2	C 23	3	3	3				
A 24	1	1	1	B 24	**2**	2	2	C 24	3	3	3				
A 25	1	1	1	B 25	**2**	2	2	C 25	3	3	3				
A 26	1	1	1	B 26	**2**	2	2	C 26	3	3	3				
A 27	1	1	1	B 27	**2**	2	2	C 27	3	3	3				
A 28	1	1	1	B 28	**2**	2	2	C 28	3	3	3				
A 29	1	1	1	B 29	**2**	2	2	C 29	3	3	3				
A 30	1	1	1	B 30	**2**	2	2	C 30	3	3	3				
A 31	1	1	1	B 31	**2**	2	2	C 31	3	3	3				
A 32	1	1	1	B 32	**2**	2	2	C 32	3	3	3				
A 33	1	1	1	B 33	**2**	2	2	C 33	3	3	3				
A 34	1	1	1	B 34	**2**	2	2	C 34	3	3	3				
A 35	1	1	1	B 35	**2**	2	2	C 35	3	3	3				
A 36	1	1	1	B 36	**2**	2	2	C 36	3	3	3				
A 37	1	1	1	B 37	**2**	2	2	C 37	3	3	3				
A 38	1	1	1	B 38	**2**	2	2	C 38	3	3	3				

A 39	1	1	1	B 39	**2**	2	2
A 40	1	1	1	B 40	**2**	2	2
A 41	1	1	1	B 41	**2**	2	2
A 42	1	1	1	B 42	**2**	2	2
A 43	1	1	1	B 43	**2**	2	2
A 44	1	1	1				
A 45	1	1	1				
A 46	1	1	1				
A 47	1	1	1				
A 48	1	1	1				
A 49	1	1	1				
A 50	1	1	1				
A 51	1	1	1				
A 52	1	1	1				
A 53	1	1	1				
A 54	1	1	1				
A 55	1	1	1				
A 56	1	1	1				
A 57	1	1	1				
A 58	1	1	1				
A 59	1	3	3				
A 60	1	1	1				
A 61	1	1	1				
A 62	1	1	1				
A 63	1	1	1				

Table S4. MS/MS parameters. Multi-reaction monitoring (MRM) transitions: Q1 MASS = precursor ion mass (amu); Q3 MASS = product ion mass (amu). TIME = dwell time. DP (V) = declustering potential (amu). EP (V) = entrance potential. CE (V) = collision energy. CXP (V) = cell exit potential.

ID	Q1 MASS	Q3 MASS	TIME	DP(V)	EP (V)	CE (V)	CXP (V)
CBD-THC-CBC	315.1	193.2	50	83	5	27	6
	315.1	135.1	50	83	5	25	9
CBG	317	193.2	50	9	10	23	6
	317	123.1	50	9	10	40	8
CBN	311.1	241.2	50	90	12	28	9
	311.1	195.2	50	90	12	34	6
CBDV	287.2	165	50	21	7	27	15
	287.2	231	50	21	7	24	17
CBGA	361.3	343.3	50	25	9	13	11
	361.3	219	50	25	9	41	11
CBDA-THCA	359.2	341.1	50	20	9	17	6
	359.2	219	50	20	9	39	9
THC D3	318.3	196.1	50	30	5	29	10
	318.3	123	50	30	5	41	10

Table S5. Limit of quantification (LOQ), limit of detection (LOD), calibration curve equation and correlation coefficient obtained in analytical procedure validation.

**Analyte**	**LOQ (ng*mL^{-1})**	**LOD (ng*mL^{-1})**	**Calibration Curve**	**R^2**
CBD	1.4	0.4	y=0.03643x+0.14657	0.99927
THC	1.6	0.5	y=0.04918x+0.21861	0.99599
CBC	2.1	0.6	y=0.03036x+0.10489	0.99873
CBDV	1.1	0.3	y=0.04820x+0.14616	0.99895
THCV	0.9	0.3	y=0.04945x+0.19749	0.99801
CBG	0.9	0.3	y=0.14858x+0.45925	0.99884
CBN	1.3	0.4	y=0.02082x+0.06200	0.99941
CBGA	2.2	0.7	y=0.06204x+0.10865	0.99856
CBDA	0.9	0.3	y=0.11645x+0.07265	0.99691
THCA	3.0	1.0	y=0.13723x+0.10739	0.99573

Table S6. The accuracy results, reported in percentage (%), obtained in analytical procedure validation.

**Analyte**	**Accuracy %**		
	**(3 ng*mL^{-1})**	**(50 ng*mL^{-1})**	**(250 ng*mL^{-1})**
CBD	118	107	101
THC	112	101	97
CBC	107	98	100
CBDV	85	102	103
THCV	126	100	98
CBG	114	96	102
CBN	122	105	103
CBGA	120	105	99
CBDA	121	98	102
THCA	120	104	98

Table S7. The intraday and interday precision results, reported in percentage (%), obtained in analytical procedure validation.

Analyte	Precision					
	Intraday (RSD)			Interday (RSD)		
	(3 ng*mL^{-1})	(50 ng*mL^{-1})	(250 ng*mL^{-1})	(3 ng*mL^{-1})	(50 ng*mL^{-1})	(250 ng*mL^{-1})
CBD	8	7	10	12	5	4.6
THC	3	7	9	3	9	8
CBC	5	7	5	7	9	13
CBDV	12	14	13	15	7	9
THCV	13	15	13	9	13	16
CBG	8	6	6	7	9	8
CBN	9	16	9	16	12	11
CBGA	8	7	9	13	11	16
CBDA	17	20	12	8	17	15
THCA	14	7	9	9	7	11
